# Cav1.4 dysfunction and congenital stationary night blindness type 2

**DOI:** 10.1007/s00424-021-02570-x

**Published:** 2021-07-01

**Authors:** Alexandra Koschak, Monica L. Fernandez-Quintero, Thomas Heigl, Marco Ruzza, Hartwig Seitter, Lucia Zanetti

**Affiliations:** 1 Institute of Pharmacy, Pharmacology and Toxicology, Center for Chemistry and Biomedicine, University of Innsbruck, Innrain 80-82/III, 6020 Innsbruck, Austria; 2 Institute of General, Inorganic and Theoretical Chemistry, Center for Chemistry and Biomedicine, University of Innsbruck, Innrain 80-82/III, 6020 Innsbruck, Austria

**Keywords:** Calcium channel, Cav1.4, Channelopathy, Channel modulation, Retinal disease, Congenital stationary night blindness type 2

## Abstract

Cav1.4 L-type Ca^2+^ channels are predominantly expressed in retinal neurons, particularly at the photoreceptor terminals where they mediate sustained Ca^2+^ entry needed for continuous neurotransmitter release at their ribbon synapses. Cav1.4 channel gating properties are controlled by accessory subunits, associated regulatory proteins, and also alternative splicing. In humans, mutations in the *CACNA1F* gene encoding for Cav1.4 channels are associated with X-linked retinal disorders such as congenital stationary night blindness type 2. Mutations in the Cav1.4 protein result in a spectrum of altered functional channel activity. Several mouse models broadened our understanding of the role of Cav1.4 channels not only as Ca^2+^ source at retinal synapses but also as synaptic organizers. In this review, we highlight different structural and functional phenotypes of Cav1.4 mutations that might also occur in patients with congenital stationary night blindness type 2. A further important yet mostly neglected aspect that we discuss is the influence of alternative splicing on channel dysfunction. We conclude that currently available functional phenotyping strategies should be refined and summarize potential specific therapeutic options for patients carrying Cav1.4 mutations. Importantly, the development of new therapeutic approaches will permit a deeper understanding of not only the disease pathophysiology but also the physiological function of Cav1.4 channels in the retina.

## Introduction

Cav1.4 L-type Ca^2+^ channels (LTCC, Cav1 family) are predominantly expressed in retinal neurons, particularly at the photoreceptor terminals. The LTCC identity in bipolar cells specifically is controversial but the presence of all Cav1 subunits has been reported including Cav1.4 [14, 80, 117]. Cav1.4 expression has further been reported in dorsal root ganglia neurons, mast cells, and T-lymphocytes (for review, see [70]).

**Fig. 1:**
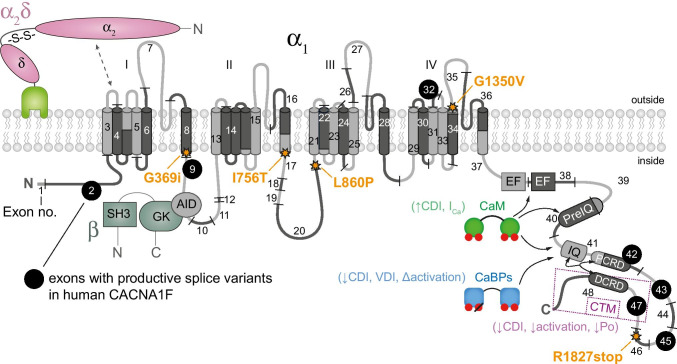
Protein topology of human Cav1.4 with coding exons, alternatively spliced exons (see Table 2) and selected mutations annotated (according to human reference sequence NP_005174) together with an intracellular β and an extracellular α2δ and potential interaction sites with calmodulin (CaM) and Ca^2+^ binding proteins (CaBP). Abbreviations: VDI, voltage-dependence of inactivation, CDI, Ca^2+^-dependence of inactivation; P_o_, open probability, AID, alpha-interaction domain; EF, EF-hand motif; other abbreviations, see main text

The gating properties of Cav1.4 channels [11, 53, 64] are perfectly suited to mediate sustained Ca^2+^ entry needed for continuous release of neurotransmitters at photoreceptor ribbon synapses in the dark [84, 102]. Upon light absorption in the photoreceptor outer segments, the closure of cGMP-gated cation channels hyperpolarizes the photoreceptors cells (below − 55 mV [110]). In the dark, photoreceptor membrane potential depolarizes (− 36 to − 40 mV) and thereby enhances tonic neurotransmitter (glutamate) release [24]. For such tonic release, only a few channels that activate rapidly at relatively negative voltages (< − 40 mV, [10, 34, 101]) and inactivate slowly are needed [10]. Indeed, heterologously expressed Cav1.4 LTCCs show fast activation and allow Ca^2+^ influx at membrane potentials negative to − 40 mV. In addition, Cav1.4 currents show slow voltage-dependent inactivation (VDI) with complete absence of Ca^2+^-dependent inactivation (CDI) [53, 84]. Of note, near physiological temperatures, inactivation kinetics were accelerated but the window current that may be seen as a “window” of voltages where the activation and steady-state inactivation curves overlap is still preserved [74]. Cav1.4 channels lack CDI due to active suppression by an inhibitory domain in their C-terminus [84, 102]. This phenomenon is referred to as C-terminal modulation (CTM). In Cav1.4 (and also Cav1.3) channels, the modulation is attributable to an interaction of a proximal (PCRD) and a distal (DCRD) C-terminal regulatory domain (Fig. 1), which are putative α-helices [84, 85]. Due to the competition of the distal C-terminus with calmodulin (CaM) binding [57], CDI is absent (or weaker in Cav1.3) [20, 84, 85]. The CTM not only determines CDI but also affects the channel’s activation gating properties and the open probability [16, 44, 45, 85]. Interestingly, CTM elimination in Cav1.3 channels in hair cells and chromaffin cells affected CDI but not the channels’ activation threshold [81].

CaM is important for Cav1.4 function because it increases current density and slows down VDI [32] in addition to its role as a specific channel-bound Ca^2+^ sensor that is tethered to upstream regions of the C-terminal tail such as the helical IQ domain adjacent to the so-called EF-hand motif (Fig. 1). Ca^2+^-binding protein 4 (CaBP4) competes with CaM for the IQ motif of the channel by disrupting interaction between IQ and the distal C-terminus [72]. Moreover, CaBP4 increased channel availability in a heterologous expression system, by increasing the channel’s window current [82]. Auxiliary subunits α2δ and β have been shown to modulate electrophysiological properties as well as increase the number of voltage-gated Ca^2+^ channels on the membrane (see review [19, 27]). The β subunit interacts with the alpha-interaction domain (AID) of the channel, while α2δ subunits mainly interact with an extracellular loop in domain I (Fig. 1). More specifically, the β2 variants β2a and β2X13, which have been reported to assemble with Cav1.4 in photoreceptors, differentially modulated Cav1.4 properties but both support slow inactivation [56] and are necessary for forward trafficking. Among α2δ subunits, α2δ4 is part of the Cav1.4 channel complex in photoreceptors [56] and supports Cav1.4 functional expression not only in tsa-201 cells [7] but also in photoreceptor terminals [49] likely by enhancing the stability of Cav1.4 channels by suppressing their turnover.

The important role of Cav1.4 in the retina is evident from mutations in the *CACNA1F* gene encoding Cav1.4 LTCCs that cause retinal diseases in humans (OMIM 300,071, 300,476, 300,600) such as congenital stationary night blindness type 2 (CSNB2, OMIM 300,071; Table 1). Mutations in the α2δ4 subunit (*CACNA2D4* gene [115]) and CaBP4 [122]—both are proteins that interact with Cav1.4 (Fig. 1)— are also associated with CSNB2 [124]. CSNB2 patients show variable levels of night blindness together with myopia, nystagmus, strabismus, and low visual acuity [15]. In particular, patients that carry *CACNA1F* mutations may present with only few or even no night vision problems [66, 124]. Visual fields in CSNB2 patients are normal but daylight vision, color vision, and visual acuity can be affected [15]. More than 50% of the patients suffer from photophobia [15], often seen in cone dysfunction syndromes [2]. Due to the phenotypic variability seen in CSNB2 patients, the only diagnostic tool is the electroretinogram (ERG; see also the “Functional phenotyping of Cav1.4 related diseases” section).

**Table 1:**
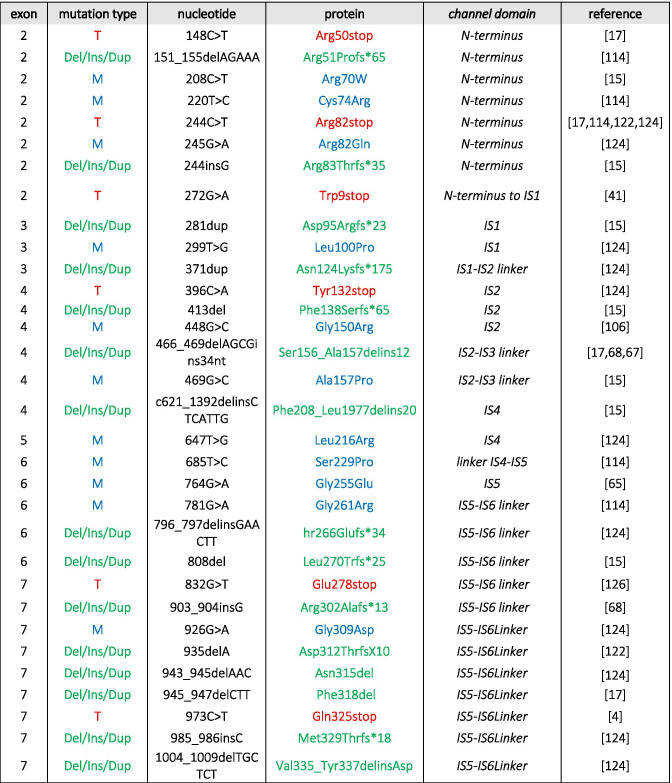

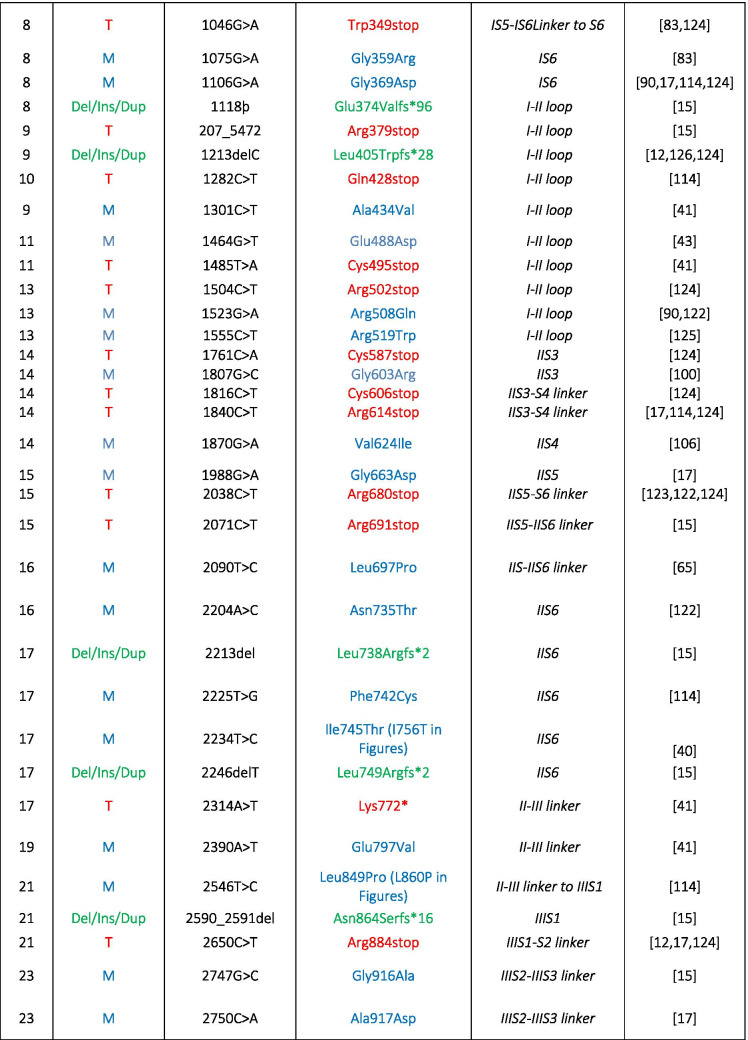

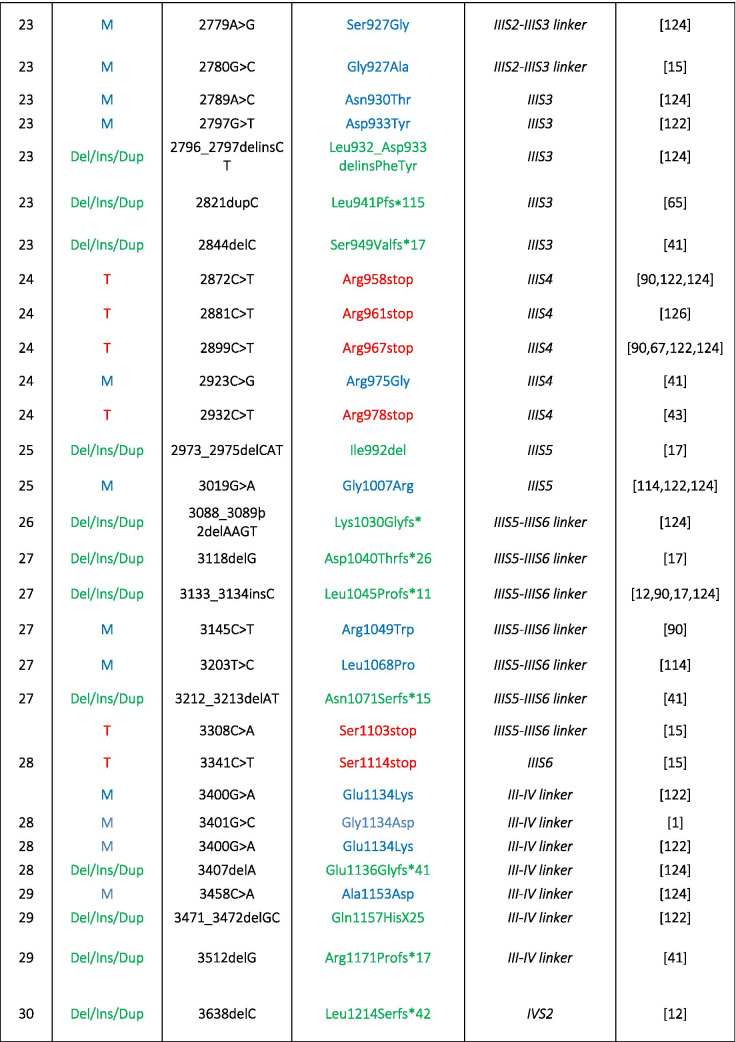

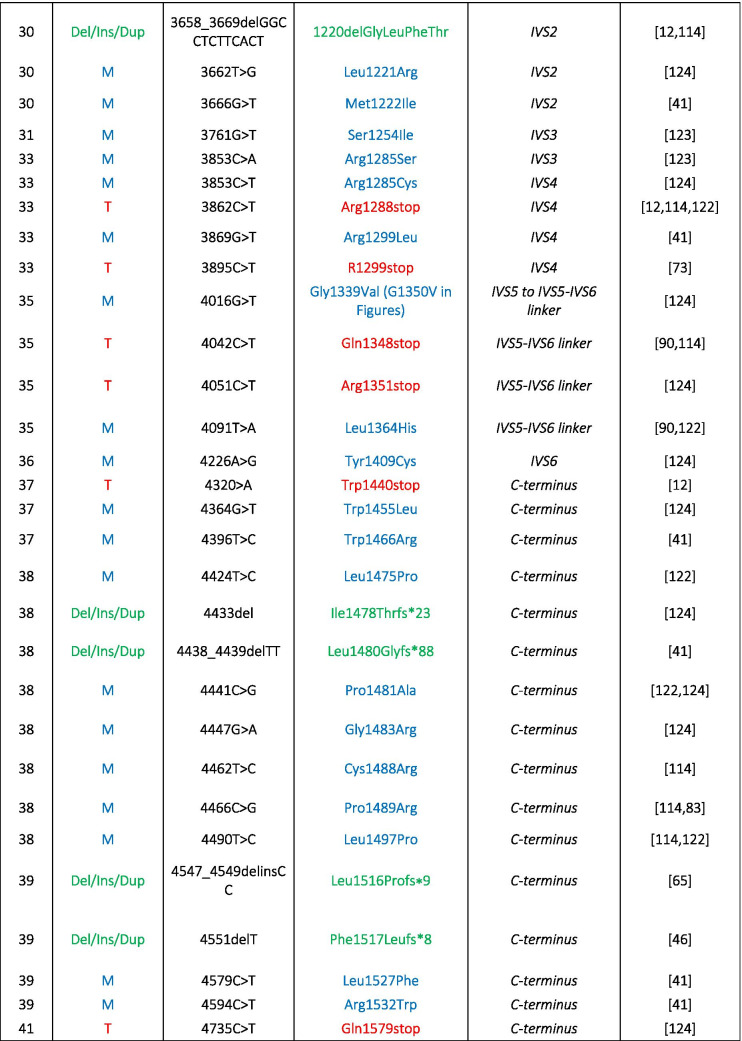

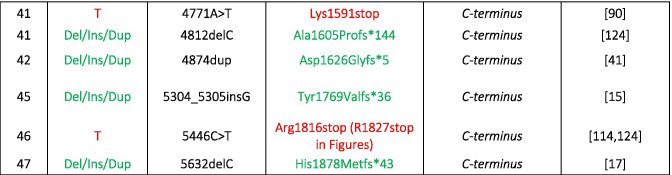
Disease-causing mutations in human Cav1.4 α1-subunits. Different types of mutations have been reported: *M*, missense; *T*, truncation; *Del*, deletion; *Ins*, insertion; *Dup*, duplication. Numbering according to reference sequence AJ006216. Note, the position 745 of Ile to Thr mutation refers to the original report [35]; the reference sequence contains a short exon 9

In this review, we focus on mutations in the *CACNA1F* gene as this gene is most commonly affected in CSNB2 patients and their differential effect on retinal visual pathways. We challenge a previously used classification of mutation types and the functional phenotyping strategies currently available. Moreover, we elucidate the influence of different splice variants on Cav1.4 dysfunction and how they might add additional functional impact, and we discuss potential specific therapeutic options for CSNB2 patients.

## Role of Cav1.4 channels

Our understanding of the role of Cav1.4 in the retina largely stems from various mouse models. Up to date, 6 different mouse models are available: two knock-out (KO) models: Cacna1fΔEx7 and Cacna1fΔEx14-17, firstly described in [63, 86]; two spontaneous insertions of a transposable element in exon2 or in the intron of exon 13–14 (nob2 [21] and nob9 mice [25]); the knock-in of the single gain of function point mutation Cav1.4 Ile756Thr (I756T; called Ile745Thr in the original report, corresponding to reference sequence AJ006216, [86]) and a most recently reported non-transmitting G369i knock-in mouse [61].

**Fig. 2:**
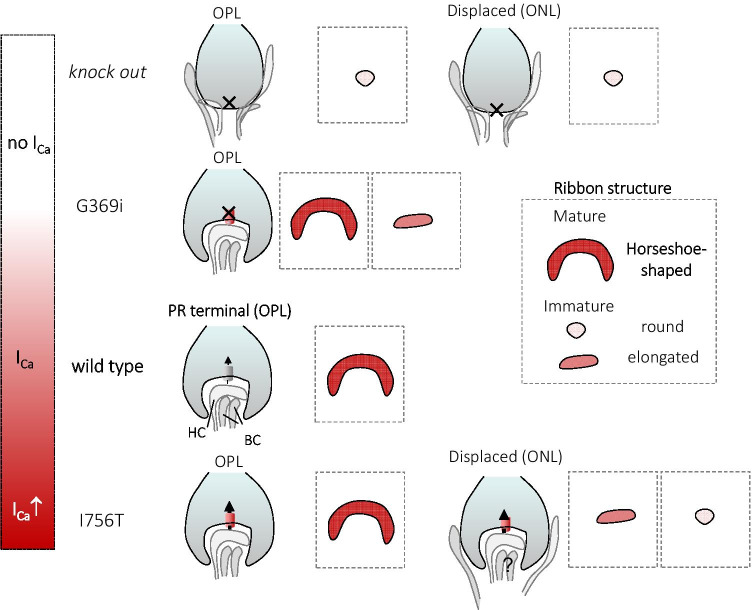
Dual role of Cav1.4 channels in the photoreceptor synapse. Presynaptic Cav1.4 channels serve as sources of Ca^2+^ ions and play a role as synaptic organizer protein. Ca^2+^ influx (I_Ca_) changes according to the Cav1.4 mutation (KO, loss-of-function [58]; G369i, non-conducting [61]; I756T, gain-of-function [35]) and exerts different structural effects: left: the presence of sprouting second order neurons (BC, bipolar cell; HC, horizontal cell) and right: changes in the ribbon structure. The inset shows the lateral view on mature (horseshoe-shaped) and immature (round or elongated) ribbons. In knock-out and I756T retinas synaptic terminals were not only located in the outer plexiform layer (OPL) but were also displaced in the outer nuclear layer (ONL) [51, 58, 120]

**Fig. 3:**
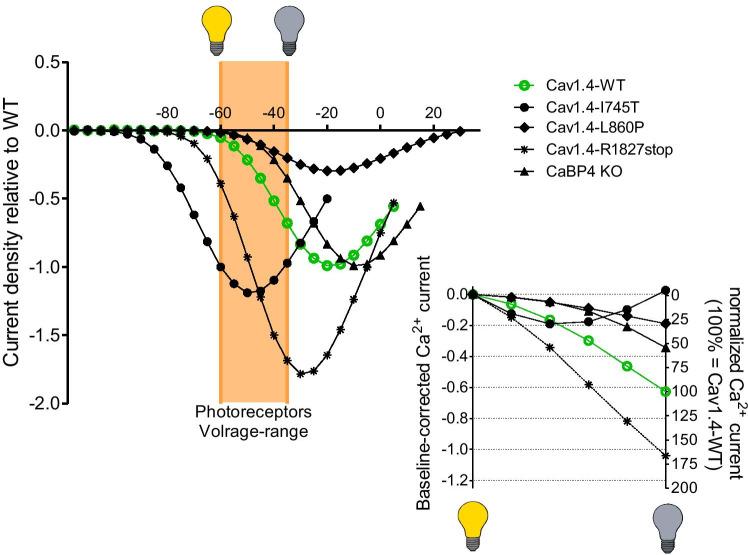
Cav1.4 mutations lead to a change in the Ca^2+^ dynamics within the photoreceptor voltage range. The current density of different Cav1.4 CSNB2-mutation relative to wild-type Cav1.4 channels (Cav1.4-WT, green) is indicated. Ca^2+^ currents measured in rod photoreceptors are the reference for the voltage dependence of Cav1.4 wild-type currents ([5]). Current densities were taken from literature: wild type: open circles, 2 mM Ca^2+^ [20] (set as − 1); I756T: filled circles, 5 mM Ca^2+^ [35]; L860P: diamonds, 15 mM Ca^2+^ [20]; R1827stop: stars, 2 mM Ca^2+^ [20]; CaBP KO: triangles, 20 mM Ca^2+^[72]. The photoreceptor voltage range is highlighted in orange: from ~  − 60 mV upon light on to ~  − 35 mV at light off. The inset shows currents that were baseline corrected to highlight the change in Ca^2+^ influx within the photoreceptor voltage range

All those animal models showed that in the absence of functional Cav1.4 channels photoreceptor ribbon synapses remained mostly immature, as evidenced by their roundish (sometimes elongated) appearance (Fig. 2, [58, 61, 77, 79, 118]). In the case of changes in the dynamics of Ca^2+^ influx, e.g., in I756T mice which carry a gain-of-function Cav1.4 channel mutation [35]. The maturation of photoreceptor synaptic ribbons is disturbed and comes along with free-floating ribbons (Fig. 2). The integrity of other proteins of the ribbon and the arciform density are compromised accordingly in this mouse model [79, 86]. The non-transmitting G369i knock-in model, however, taught us that although leading to shorter ribbons, the presynaptic assembly of rod synapses can proceed without Cav1.4 meditated Ca^2+^ signals (Fig. 2, [61]). Together, those data support a dual role of presynaptic Cav1.4 channels: they serve as a source of Ca^2+^ ions and play an important role as synaptic organizer proteins in the synapse of the visual pathway. Accordingly, Cav1.4 dysfunction leads to postsynaptic changes like sprouting of bipolar and horizontal cell dendrites (Fig. 2) comparable to the KO phenotype of the protein bassoon, which links the ribbon and the arciform density/plasma membrane compartment containing Cav1.4 channels (for review [78]). Moreover, reduced amounts of appropriate synaptic scaffolds, such as PSD-95 in the KO retina [63], may not only limit the retention of Cav1.4 channels in the presynaptic membrane but also affect the correct positioning of postsynaptic proteins. Sprouting is always seen in case of a pronounced change in Ca^2+^ influx (either KO or gain of function I756T). But whenever Cav1.4 protein was present (at least some), invaginating contacts have been preserved (Fig. 2). Synaptic defects might therefore not only correlate with the extent to which presynaptic Cav1.4 channels are lost (this is also the case when auxiliary subunits of Cav1.4 LTCCs are missing [49, 105]) but also with Ca^2+^ dynamics (see below, Fig. 3).

## Spectrum of Cav1.4 mutations

Variations in the clinical manifestation of CSNB2 might arise from the different types of Cav1.4 mutations causing different channel defects. Various studies in heterologous expression systems showed that the spectrum of Cav1.4 dysfunction is indeed wide [20, 35, 37, 38, 64, 74, 84].

So far, we framed Cav1.4 mutations in the canonical categories “gain-of-function,” “loss-of-function,” and “CTM-function impaired” [89]. Nevertheless, while from a biophysical point of view a higher current density is a clear gain of channel function (and vice versa), the definition does not reflect what happens in the (mouse) retina. For example, in I756T (gain-of-function: hyperpolarizing shift of the half-maximal voltage of activation (V_½_) and higher current density; circle in Fig. 3) and the nob2 (loss-of-function: reduction of channel surface expression, comparable to the previously characterized L860P, diamond in Fig. 3), retinas are not only virtually undistinguishable from a morphological point of view, but also their ERGs are comparable as scotopic and photopic b-wave are reduced to a similar extent [21, 50]. It seems that more than the amount of Ca^2+^ influx, the relative change of Ca^2+^ (ΔCa^2+^) within the photoreceptor voltage range (Fig. 3) is an important readout. Mimicking a loss of function mutation, also the CaBP4 KO mouse was validated as a model for CSNB2 [72]. In vitro, co-expression of Cav1.4 with CaBP4 leads to a 10 mV hyperpolarizing shift compared to Cav1.4 alone [72, 82]. In photoreceptors lacking CaBP4, this would mean a reduction of Δ Ca^2+ ^influx within the photoreceptor voltage range of about 50% (up-triangle in Fig. 3) that could explain the CSNB2 phenotype. Burtscher and colleagues characterized another human Cav1.4 mutation leading to an impairment of the CTM, named R1827stop. This mutation not only shows gain-of-function features but also changes the channel inactivation properties unmasking CDI due to the loss of functional CTM [20]. Mutations that affect the CTM will rather not support continuous Ca^2+^ influx [20] and might thereby reduce the dynamic range of photoreceptors. Truncations downstream of the CDI machinery in the Cav1.4 C-terminus—physiologically by alternative splicing [95] or introduced by an artificial mutation [84, 102]—are catching our further interest (see also the “Regulation of Cav1.4 function by alternative splicing” section). In addition to a more hyperpolarized activation voltage, Cav1.4-R1827stop shows increased current density due to the higher open probability of the channel [16, 20]. Of note, the mutation does not affect the unitary conductance of the channel [16, 20] but whether the number of channels expressed in the plasma membrane is increased has not yet been investigated in any of the Cav1.4 gain-of-function mutations reported. Together these gating changes are resulting in a change in Δ Ca^2+ ^(asterisks in Fig. 3). Since we are lacking data from in vivo models, it is still unclear which of these processes dominates the phenotype of Cav1.4 mutants with impaired CTM function. Thus, this peculiar mutation will be worth being studied in retinal photoreceptors to gain better understanding of how changes in Ca^2+^ dynamics affect this ribbon synapse.

## Functional phenotyping of Cav1.4 related diseases

ERGs of CSNB2 patients show that both, scotopic and photopic responses, are affected. Patients present with an abnormal dim scotopic ERG and a typical negative bright-flash ERG with large a-waves, but severely reduced b-waves; oscillatory potentials are missing. This ERG phenotype, in particular the reduction in the b-wave in both scotopic and also photopic ERG, highlights the transmission problem from rod and cone photoreceptors to bipolar cells (for review, see [124]).

It is a common scientific agreement that Cav1.4 α1 together with the accessory β 2 and α2δ4 subunits form the major LTCC complex at the photoreceptor terminal (Fig. 1) that mediates Ca^2+ ^influx and consequently regulates glutamatergic vesicle release in both rods and cones. While in all KO models of the abovementioned subunits the b-wave was almost completely absent in both scotopic and photopic ERG recordings [9, 103, 105], in other CSNB2 mouse models the readout was more complicated. In fact, a body of literature highlights the differences between rod and cone photoreceptors in retinal disease [29, 49, 61, 105, 118]. In particular, the cone morphology appears to be less affected in CSNB2 models compared to rods [8, 49, 105, 118]. However, the photopic ERG is also severely affected (for review, see [124]). For mutations that lead to changes in the gating properties of the channels the reason may be found in different mechanisms to cope with intracellular Ca^2+^ concentration (Fig. 3, but see also [48]). Still, further important information regarding specific retinal (dys) function might come with ERG protocols better suited than common ones to investigate separately different pathway (e.g., ON, OFF, [96]). This differentiation would be important because de- and hyperpolarizing bipolar cells do not only express different postsynaptic receptor pools but also take different positions in the triad synapse (e.g., OFF bipolar cell show non-invaginating, flat contacts) and might therefore be differentially affected by presynaptic mutations. In the nob2 mouse model, where only 10% of Cav1.4 full-length transcript is predicted to be expressed, both light-adapted and dark-adapted ERGs are present [21]. Of note, Chang and colleagues implemented their ERG finding with more sensitive extracellular in vivo single-unit retinal ganglion cell recordings which showed a lower spontaneous activity and a change in the gain of their light response in ON ganglion cells whereas OFF ganglion cell responses were actually unaffected. Thus, we need more appropriate diagnostic tools because the relative preservation of the OFF pathway is most likely undetected in the ON-dominant ERG flash as is the influence of other pathways in the dark-adapted ERG. This is supported by another recent study that emphasized the different effects of a Cav1.4 mutation in the scotopic and photopic retinal pathways. In fact, in I756T mice, the cone-cone bipolar cell transmission was severely affected whereas the rod pathway was still responding to light [120].

Moreover, in the non-transmitting G369i mutant in which the morphology of the ribbon synapse is largely maintained the ERG highlighted a positive b-wave at high light intensities [61]. By contrast, no discernible b-wave can be detected in Cav1.4 KO mice [67], comparable to mice completely lacking rod and cone phototransduction [23], ruling out intrinsically photosensitive ganglion cells as the source of this ERG b-wave. This finding suggests that (i) cones might express also other Ca^2+^ channels in addition to Cav1.4 and (ii) some residual vision might be present and is worth investigating. It is therefore imperative to solve the channel (subunit and/or splice variant) composition of rods and cones to be able to better understand CSNB2 pathology.

## Regulation of Cav1.4 function by alternative splicing

Alternative splicing is a common feature of voltage-gated Ca^2+^ channel subunits thought to confer an increased diversity to the biophysical properties and potential interactions of the channel. Among α1, the extent of Cav1.2 and Cav1.3 alternative splicing also correlates with their widespread tissue distributions (including heart, vascular smooth muscle, endocrine cells, and neurons in the nervous system) compared to the narrow expression patterns of Cav1.1 (largely specific for skeletal muscle) and Cav1.4 (mainly in the retina). This difference suggests potentially less necessity for differential tuning of channel properties for the latter two isoforms. Nonetheless, several Cav1.4 splice variants have been determined in mRNA isolated from the human retina and some of them have been functionally characterized (Fig. 1, Table 2, [33, 95]).

**Table 2:** Productive Cav1.4 splice variants found in human retina. We excluded splice variants that can be considered non-productive (inducing frameshifts and premature stops or otherwise leading to deletions of transmembrane segments, including the double frameshifts around exons 16–18). The % of each variant at respective locus was taken from Tan et al. (2012) [95] (#) and Haeseleer et al. (2016) [33] (##). Changes in activation and inactivation properties are provided where data is available. Data sources: #1 [95] (Cav1.2 chimera), #2 [95], ## [33], ### Liu et al. (2017) [59] (Cav1.3 chimera); *V*_*½*_, shift in the half-maximal voltage of activation; *VDI*, voltage-dependence of inactivation; *CDI*, Ca^2+^-dependence of inactivation

Splice variant	% at locus	Structural change	Functional characterization	
			Activation (V½)	Inactivation
Ex2x	3.4% ^#^	N-terminal sequence changed (mutually exclusive in-frame exon)	n.a	n.a
Ex9d-	9.1% ^#^	shorter I-II linker, preserved AID (in-frame alternative splice donor site; ΔGSMAEEGRAGH)	n.a	n.a
ΔEx32	17.9% ^#^	shorter IVS3-IVS4 linker (in-frame exon skipping; ΔNGGHLGE)	← ← (− 7 mV) ^###^	n.d.^###^
Ex42d +	n.a	C-terminal sequence changed; longer C-terminus (in-frame alternative splice donor site; + VGTSFHSPRNLI)	← (−2.6 mV) ^#1^	unchanged^#1^
Ex43*	13.6% ^#^	C-terminal sequence changed; shorter C-terminus, CTM deleted (novel exon with stop; + SRDEVLPCWPGWFRTPDLR)	← ← (− 12.5 mV) ^#2^	VDI↓ CDI↑↑^#2^
Ex45a-	12.7% ^#^ 13.8% ^##^	C-terminal sequence changed; shorter C-terminus (in-frame alternative splice acceptor site; ΔLSYLDEQAGTPPCSVLLPPHR)	→ (1.2 mV) ^#1^ ← (− 3.0 mV) ^##^	unchanged^#1, ##^
ΔEx47	< 2% ^##^	proximal CTM (PCRD) deleted (in-frame exon skipping; ΔGSWATPPQRGRLLYAPLLLVEEGAAGEGYLGRSSGPLRTFTCLHVPGTHSDPSHGKRGSADSLVEA)	← ← (− 8.0 mV) ^##^	VDI normal, CDI↑^##^

Unsurprisingly, the variants leading to substantial deletions in the C-terminus including the CTM (Ex43* and ΔEx47) exhibit pronounced CDI, likely through facilitated CaM binding to the IQ domain region (Fig. 1, [108]). In addition, both variants also impose a marked hyperpolarizing shift of their V_½_. The abundantly expressed Ex45a- (called Δex p45 in [33]), with only a minor shortening of the C-terminal sequence, has little to no effect on the current–voltage (I-V) relationship or CDI properties. Similarly, the Ex42d + variant exhibits little change relative to the canonical variant’s biophysical properties; however, this variant has only been studied in a chimeric channel [95]. Both Ex45a- and Ex42d + don’t affect the putative PCRD/DCRD sequences, thus no effects on CDI are expected. The ΔEx32 variant in the IVS3-IVS4 linker has also only been studied in a chimera but exhibited a substantial hyperpolarizing shift of the I-V curve (effects on VDI or CDI were not tested, [59]). Of note, pharmacological properties can be modulated by alternative splicing, seen for example in the sensitivity of C-terminal Cav1.3 splice variants for dihydropyridines (DHP) [42]. There is no published data on pharmacological properties of different Cav1.4 splice variants to date.

The interplay between mutations and splice variants is an important aspect for the understanding of the functional impact that splicing might have on a mutated channel. Thus far the effect of splice variants on the function of channels harboring a mutation and the pathology caused has been largely understudied. An example of a systematic comparison has been performed by Hofer and colleagues [39], where the biophysical properties of the S652L mutation in Cav1.3 were compared between the full-length channel and a short variant, lacking a large part of the C-terminus, including the Cav1.3 CTM. Most mutation-induced changes were similar in both variants; however, CDI was reduced only in the short variant. The only study of alternative splicing with a mutation in Cav1.4 investigated splice variant-dependent effects of the I756T mutation by comparing the mutation effect on canonical full-length Cav1.4 with the variant lacking exon 47 (ΔEx47) [109]. The authors found comparable hyperpolarizing shifts of the V_½_ induced by the I756T mutation in full-length (− 20 mV) and ΔEx47 (− 19 mV) channels but a substantial reduction of current density only for ΔEx47 (~ 75%), with effects of the splice variant also on current kinetics, especially on the time constant of deactivation. The interesting aspect of these splice variant-dependent effects of the mutation lies in the cumulative nature of the V_½_ change, leading to an additive hyperpolarizing shift that results in a very negative activation threshold for I756T-ΔEx47. Consequently, mutations can not only have different effects on channel properties depending on the splice variant but also changes due to splice variant and mutation can be cumulative and thus cause an exacerbation of the effects.

Interestingly, some effects might not just be modulated by the splice variant but can indeed only affect specific splice variants, for example when a mutation is localized inside a cassette exon or inside one of several mutually exclusive exons. For example, Cav1.2 mutations in patients with Timothy syndrome (TS) can be localized in either one of its mutually exclusive exons 8a (TS1) or 8 (TS2), leading to specific effects depending on whether the mutation-carrying exon is expressed in the tissue of interest [87]. Of the Cav1.4 mutations published so far, this effect can potentially occur with mutations in exon 2 for which an alternative exon exists (Ex2x, [95]) and is therefore likely not of major concern for the understanding of pathological changes of Cav1.4 channel function.

## Regulation of Cav1.4 function by subunit composition

What is of high relevance for our understanding of functional changes imposed by mutations is the auxiliary subunit composition of Cav1.4 channel complexes. Luckily, there seems to be limited diversity in auxiliary subunit expression in retinal photoreceptors with both rods and cones expressing α2δ4 and β2. For both of these isoforms, there are limited reports on alternative splicing, with one study showing a truncating variant of α2δ4 in the retina [7] and another study detecting the expression of a subvariant of β2a, called β2X13, that differs in the inclusion of exon 7B instead of the common exon 7A [56]. Most importantly, a recent publication showed different biophysical properties of the I756T mutation in channel complexes made from β2a and α2δ1 in comparison with the published native composition with β2X13 and α2δ4 [109]. While parameter changes were qualitatively the same in both complex compositions, mainly a marked hyperpolarizing shift of the V_½_ induced by the I756T mutation, the magnitudes were different and distinctly dependent on Cav1.4 splice variant. In particular, a difference in the activation gating between full-length Cav1.4 and ΔEx47 carrying the I756T mutation was only apparent with β2X13 and α2δ4. Crucially, the loss of Ca^2+^-selectivity in I756T-mutated Cav1.4 that they observed was dependent on co-expression with β2X13 and α2δ4 and was absent with β2a and α2δ1 [109].

There is to date no definite proof for cell-type specific splicing of Cav1.4 and/or auxiliary subunits in rods versus cones (or bipolar cells), which has several important consequences. Distinct splice variant-dependent mutation effects could, however, have a deeper impact on one of the cell types. One piece of evidence for a differential expression of Cav1.4 splice variants might be found from pharmacological studies. Neuromodulators like nitric oxide, somatostatin, or dopamine modulate Ca^2+^ currents in rod and cone photoreceptors differently [3, 54, 88], which could derive from a difference in the composition of Ca^2+^ channel complexes or Cav1.4 splice variants.

In summary, the definitive impact of a Cav1.4 mutation is dependent on the auxiliary subunit composition of the channel complex and on the Cav1.4 splice variant that is expressed. Until now, we only know of the existence of splice variants but we do not know if several variants are co-expressed by some cell type(s) or whether each variant has a distinct cell type of origin. There is clearly a need to determine the Cav1.4 splice variant expression in different retinal cell types to lay the foundation for an understanding of how disease-causing mutations would be modified by this factor. In heterologous expression systems, we should then consider the potential variability due to the Cav1.4 splice variant used and, in particular, also the auxiliary subunits that are co-expressed. Finally, the splice variant influence is of relevance for gene supplementation therapy approaches, where one consensus variant would be supplemented to all cell types which might not be the ideal fit for the native function in cells normally expressing an alternative Cav1.4 variant.

## Pharmacology of Cav1.4 channels

The DHP sensitivity of LTCCs varies between tissues, most likely due to not only their differential Cav and accessory protein expression but also alternative splicing of α1 subunit (see the “Regulation of Cav1.4 function by alternative splicing” section, [42]). Consistent with the Ca^2+^ channel pharmacology in photoreceptors, which previously suggested a low affinity for DHPs [107], Cav1.4 exhibits about fivefold lower sensitivity to DHPs than Cav1.2 at negative membrane potentials [52, 69, 116]. Still, compared to other LTCCs (Cav1.2/Cav1.3), the Cav1.4 pharmacology has been poorly studied and further work will be required before high-affinity Ca^2+^ channel blockers could be efficiently applied (for review, see [119]). Of note, the DHP sensitivity for mutated channels should be studied separately as some Cav1.3 variants showed different affinity to DHPs compared to the wild type [39, 76]. As a matter of fact, the I756T mutation showed a tenfold higher sensitivity to the DHP nilvadipine compared to wild-type Cav1.4 [120]. Zanetti and colleagues tried to revert the retinal phenotype caused by the gain of function mutation I756T with acute application of the drug in ex vivo retina. While this approach was not effective, in vivo long treatment with low-dose Ca^2+^ channel blockers could still be beneficial.

Instead, due to toxic side effects expected from activation of Cav1.2 and Cav1.3 in other tissues, pharmacological activation of mutated Cav1.4 channels (still expressed but with strongly reduced current density because of e.g. increased protein turnover but unchanged single channel properties [20] by LTCC activators (e.g., BayK8644)) would not be clinically applicable to humans (for review see [119]). Gating modifying drugs that would change activation and inactivation channel properties are currently not available but are on demand to optimize the dynamic range of Ca^2+^ signaling in retinal photoreceptors expressing Cav1.4 channel mutations (Fig. 3).

## Options for targeted therapies in congenital stationary night blindness type 2

A milestone that cleared a path for new therapies targeting retinal diseases in clinical trials was the approval of Luxturna ^®^, the first gene therapeutic drug used for the retinal dystrophy Leber congenital amaurosis type 2 [62]. Viral vectors as vehicles to transport genetic information into cells have been employed extensively in the gene therapeutic field [60].

Most of the current retinal gene therapies are employing recombined adeno-associated virus (rAAV) vectors due to the lack of human pathogenesis and the low immune response compared to other viral vectors [13]. However, all of the current clinical trials employing rAAVs transport small genes because the efficient packaging capacity is below 5000 base pairs [28, 112]. Thus, to circumvent the size limitation of AAVs transporting bigger genes (e.g., *CACNA1F*), different procedures such as intermolecular recombination, RNA-, and protein trans-splicing are needed [91, 97, 98].

**Fig. 4:**
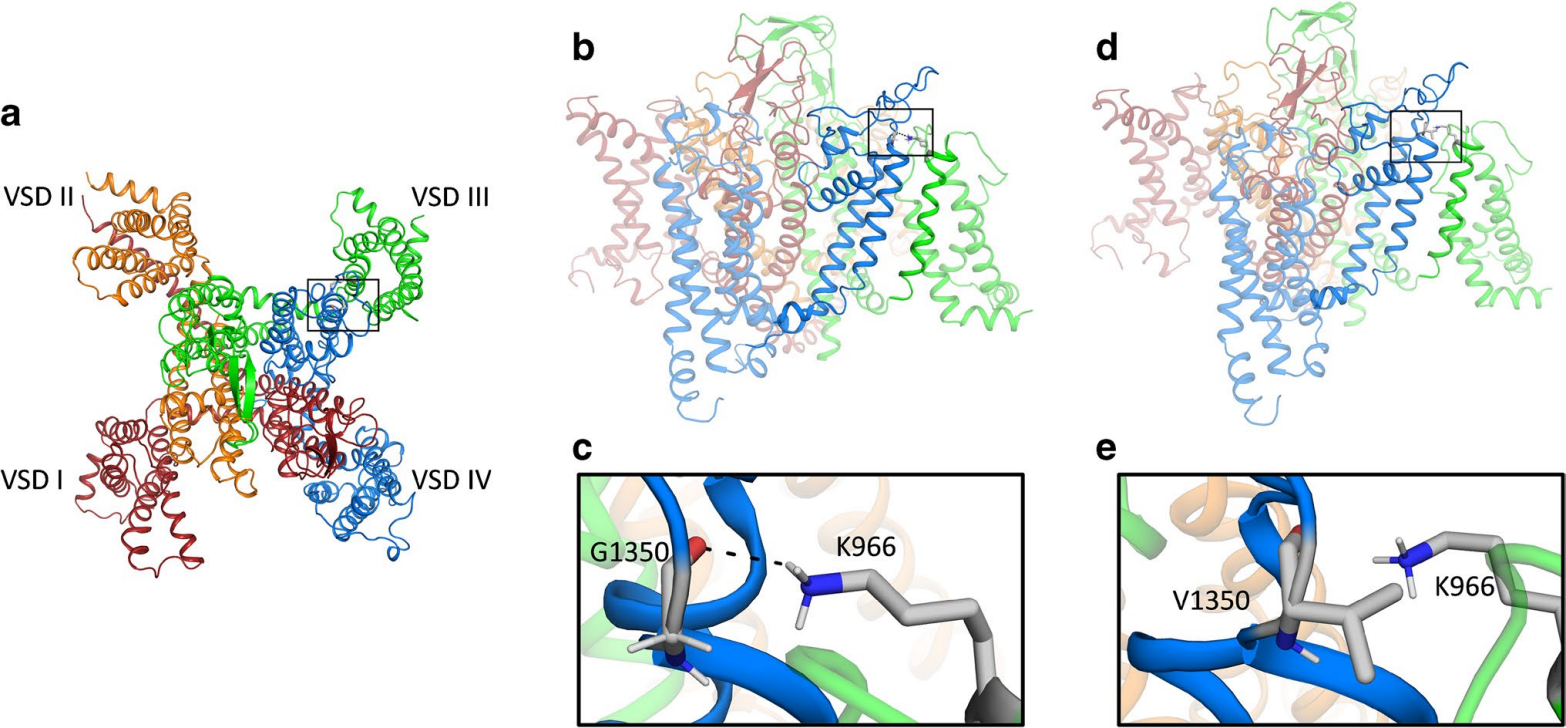
Structure model of the wild type and the G1350V mutant Cav1.4 α1 subunit. Panel **a**: top view of the Cav1.4 α1 subunit, highlighting the position of the G1350V mutation, which is located at the end of the IVS5 transmembrane helix. Panels **b** and **c** illustrate the interdomain interaction of VSD III (K966) with the pore loop of VSD IV (G1350) the wildtype Cav1.4 α1 subunit which cannot be formed between V1350 and K966 (**d**, **e**)

Cav1.4 mutations leading to fewer functional channels, due to decreased channel stability and promoted misfolding ([20], for review see [89]), would be a perfect target for augmentation gene therapy. As an example, we predicted the structural consequences of a glycine to valine mutation located extracellularly at the end of the IVS5 transmembrane helix in the voltage sensor domain (VSD) IV (G1350V) by building a homology model based on the cryo-electron microscopy structure of the Cav1.1 α1 subunit in the inactivated state (PDB accession code: 5GJW) [113]); sequence similarity with Cav1.4 α1 about 85%; Fig. 4). As G1350 is located at the beginning of the pore loop; it introduces mobility which might be required for forming the kink of the pore loop (Fig. 4b, c). The mutation might therefore destabilize a favorable interdomain interaction of the G1350 with the neighboring VSD III (Fig. 4d, e) resulting in a lower open probability and/or decrease of the stability of the channel (previously also observed for the intracellularly located loss of function mutation L860P, [20]). Interestingly, a similarly located mutation in the segment S6 of the Cav1.2 VSD I—that might also form an interaction with the pore loop—is a causal long QT syndrome mutation [30]. Such mutations would benefit from the application of chemical chaperones because the gating properties of the remaining currents are comparable to wild type ([20], Fig. 3 for L860P). A majority of mutations, however, are predicted to cause severe structural changes such that they are unlikely to form functional channels, often due to premature truncation (Table 1). Truncated Cav1.4 channels might therefore not even be expressed because nonsense-mediated mRNA decay eliminates mRNAs containing premature translation-termination codons [18].

Gene augmentation therapy, however, might not be practical for all Cav1.4 mutations. In case of mutations that cause gating changes, inhibitory RNA could be expressed in photoreceptor cells to bind to the mutation-containing mRNA and initiate RNA degradation [75, 94]. This procedure would lead to a decrease in non-functioning protein product and may be beneficial for cell survival if combined with a gene supplementation therapy.

Furthermore, errors in the splicing procedure of pre-mRNA might change the functionality of the translated protein (Table 3). Here, antisense oligonucleotides can be used to increase the likelihood of excluding or including individual exons during the splicing process [6, 31] and thereby restore a wild-type coding sequence. Lastly, an additional way to treat Cav1.4 mutations could be to correct them in the photoreceptor genome using zinc finger nucleases [99], TALENs [111], or CRISPR/Cas [36, 92] as molecular tools.

**Table 3:** Location and nucleotide position of so far uncharacterized splice site mutations

Exon or exon-exon	Nucleotide	Reference
3–4	c.382-2A > G	[96]
4–5	c.523-2A > G	[96]
17	c.2288 + 1G > A	[22]
17	c.2288 + 5G > T	[22]
19	c.2387-1G > C	[97]
20	c.2544-1G > A	[22]
20	c.2571 + 1G > C	[101]
21	c.2673 + 3G > A	[97, 98]
21	c.2674–2; 2674-3delCA	[97]
22	c.2733 + 1G > C	[101]
24	c.2938 + 1G > A	[96, 98]
24–25	c.2961 + 1G > A	[96]
28	c.3439–1 GCGTC > TGG	[47]
33	c.3942 + 2 T > C	[97]
33	c.3942 + 2 T > A	[97]
35	c.4101-1G > C	[22, 97]
39	c.4590-2A > G	[96]
40–41	c.4724-2A > G	[96]

Currently, no gene therapy for CSNB2 is available. However, Waldner and colleagues established a mouse line that showed a partial structural and functional rescue of retinal integrity by cre-induced expression of a transgenic Cav1.4 [104]. Cre expression was controlled by a Pax6 promoter, resulting in Cav1.4 expression in the early developmental stage. Of note, ERGs and visually evoked potentials in the visual cortex supported signal transduction to the brain. Immunohistochemical analysis of the transgene revealed patchy expression throughout the retina. Since the integration of the transgene into the genome should be at the same locus for all nucleus-containing cells, the patchy expression pattern hints to either epigenetic silencing or post-transcriptional degradation. The structural features of the retina resembled a mosaicism phenotype, in which columns show either the knockout or the wild-type phenotype. Michalakis and colleagues reported a similar phenotype in Cav1.4 heterozygous female mice, caused by X-chromosome inactivation [65]. Avoiding genomic silencing is of crucial interest for a retina-wide expression of the transgene. A possible strategy could be to employ the CRISPR/Cas system to guide methyl-cleaving proteins to the genomic integration site of the transgene and carry out epigenetic editing by removing the methylation labels responsible for the gene inactivation [26]. One option might be to flank the transgene with insulator sequences; in between those genomic DNA is known to be better accessible and transcriptionally active, resulting in long-term expression in vivo [22, 93]. Another option would be to target the transgene into known genetic safe harbor locations, where gene silencing is not expected (e.g., the AAVS1 locus, for review, see [71]).

Laird and colleagues, on the other hand, employed a tamoxifen-inducible promoter to temporally express transgenic Cav1.4 [55]. Rods got transfected with transgene-carrying plasmids by retinal in vivo electroporation at the day of birth because rod-precursor cells are still dividing at that developmental stage and plasmid DNA can enter the nucleus during mitosis. In both young and mature animals, induction of Cav1.4 expression partially rescued synaptic features. Most likely due to the low transfection rate of rods, which was 10%, the ERG could not be restored. Thus, the performance improvement in a visually guided water maze test was limited. Yet there are technical difficulties to be overcome because of retinal detachment observed in the tamoxifen-induced group.

Taken together, a gene therapeutic approach will bring us closer to a better understanding of the role of Cav1.4 channel for synapse plasticity and pave the way for clinical applications in human patients.
